# Self-Reported Eating Speed and Incidence of Gestational Diabetes Mellitus: the Japan Environment and Children’s Study

**DOI:** 10.3390/nu12051296

**Published:** 2020-05-02

**Authors:** Jia-Yi Dong, Satoyo Ikehara, Takashi Kimura, Meishan Cui, Yoko Kawanishi, Tadashi Kimura, Kimiko Ueda, Hiroyasu Iso, the Japan Environment and Children’s Study Group

**Affiliations:** 1 Public Health, Department of Social Medicine, Osaka University Graduate School of Medicine, Osaka 5650871, Japan; dongjy@mail3.sysu.edu.cn (J.-Y.D.); s-ikehara@pbhel.med.osaka-u.ac.jp (S.I.); saimiyoshi@163.com (M.C.); 2 Department of Public Health, Hokkaido University Graduate School of Medicine, Sapporo 0608638, Japan; kimura@med.hokudai.ac.jp; 3 Department of Obstetrics and Gynecology, Osaka University Graduate School of Medicine, Osaka 5650871, Japan; angeltears90@hotmail.co.jp (Y.K.); tadashi@gyne.med.osaka-u.ac.jp (T.K.); 4 Maternal & Child Health Information Center, Osaka Women’s and Children’s Hospital, Osaka 5941101, Japan; kimi-h-u@wch.opho.jp; 5 Department of Public Health Medicine, Faculty of Medicine, University of Tsukuba, Tsukuba 3058575, Japan

**Keywords:** eating speed, gestational diabetes, cohort study, prevention

## Abstract

There is little evidence linking eating speed to gestational diabetes mellitus (GDM) incidence. We therefore aimed to evaluate the prospective association of eating speed with GDM incidence. Overall, 97,454 pregnant women were recruited between January 2011 and March 2014. Singleton pregnant women who did not have GDM, heart disease, stroke, cancer, type 1 diabetes, and/or type 2 diabetes at the time of study enrollment were eligible. Each woman was asked about her eating speed at that time via a questionnaire. Odds ratios of GDM in relation to eating speed were obtained using logistic regression. Among the 84,811 women eligible for analysis, 1902 cases of GDM were identified in medical records. Compared with women who reported slow eating speed, the age-adjusted odds ratios (95% confidence interval) of GDM for women who reported medium, relatively fast, or very fast eating speed were 1.03 (0.90, 1.18), 1.07 (0.94, 1.23), and 1.28 (1.05, 1.58), respectively. Adjustment for demographic, lifestyle-related, and dietary factors including dietary fat, dietary fiber, and energy intakes yielded similar results. The association was attenuated and no longer significant after further adjustment for pre-pregnancy body mass index. The mediation analysis showed that being overweight accounted for 64% of the excess risk of GDM associated with eating speed. In conclusion, women who reported very fast eating speed, compared with those reporting slow eating speed, were associated with an increased incidence of GDM, which may be largely mediated by increased body fat.

## 1. Introduction

Diet is widely known to play an essential role in promoting health and preventing disease. In addition to what we eat, the way we eat may also impact our health. In particular, the effects of eating speed on obesity, as well as obesity-related diseases, have received increasing research interest over the past decade. A number of cross-sectional studies have suggested eating quickly is associated with a higher prevalence of obesity [1]. Excess energy intake is one possible explanation for the association between faster eating and risk of weight gain and obesity [2]. Emerging evidence also indicates that eating quickly may increase the risk of metabolic syndrome [3,4,5] and type 2 diabetes [6].

Gestational diabetes mellitus (GDM) is a complication affecting about 7% of pregnant women and has various influences on both mothers and their offspring [7,8,9]. Adverse pregnancy outcomes stemming from GDM include macrosomia, cesarean section, and shoulder dystocia [10,11]. Over the long term, GDM has been shown to be associated with an elevated risk of type 2 diabetes and cardiovascular diseases in mothers [12,13], and a higher risk of obesity and insulin insensitivity in their children [14,15].

Observational studies and clinical trials have suggested diet plays an important role in preventing GDM [16,17], but whether eating speed is independently associated with GDM is largely unknown. Moreover, whether body mass index (BMI) plays a mediating role and to which extent BMI may account for the possible association between eating speed and GDM are also uncertain. In the present study we used a large Japanese national birth cohort to examine the prospective association between eating speed and GDM and to test whether BMI was a mediator of the association.

## 2. Research Design and Methods

The present analysis was based on the Japan Environment and Children’s Study (JECS), launched by the Ministry of the Environment, Japan, which was primarily aimed at evaluating the effects of environmental factors on pregnancy and children’s health. Genetic, socioeconomic, and lifestyle factors were also examined. The study design is detailed elsewhere [18,19]. In brief, about 100 thousand pregnant women (median gestational age: 12 weeks) were recruited in 15 areas across Japan, from January 2011 to March 2014. We obtained information on demographic information, socioeconomic status, disease history, lifestyles, and dietary habits of each mother at the time of study enrollment via a self-administered questionnaire. 

**Figure 1:**
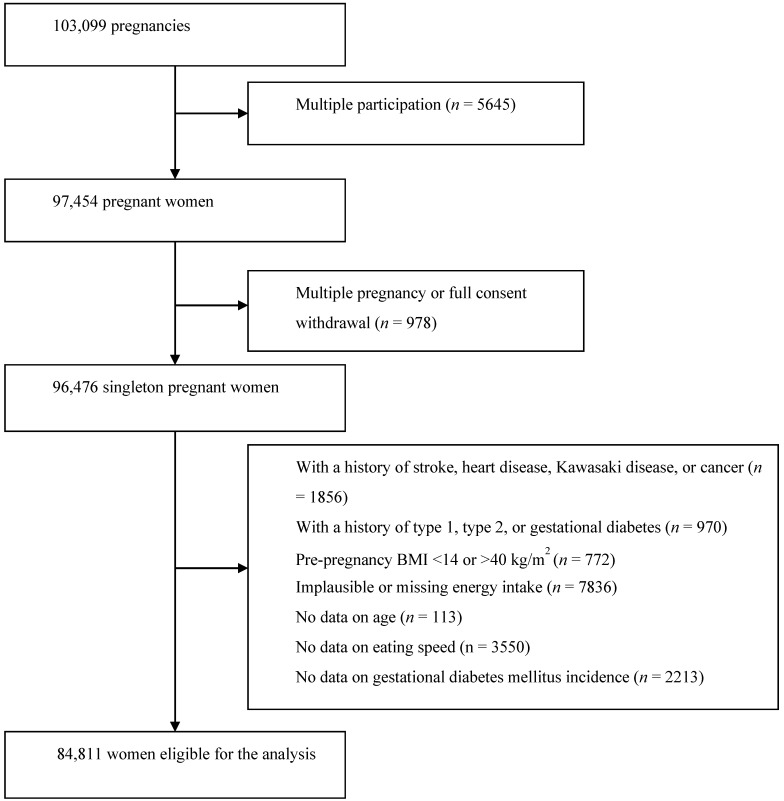
Flowchart for participant selection.

There were 103,099 pregnancies from 97,454 women recruited. Women were recruited voluntarily at the first prenatal examination and/or when they reported their pregnancies at local government offices. In 2013, the recruitment covered about 45% of pregnancies in the study area [19]. Among the 97,454 women, those who had singleton pregnancy and were not multiple participations were considered for this analysis. Women were eligible if they did not have GDM, heart disease, stroke, Kawasaki disease, cancer, type 1 diabetes, and/or type 2 diabetes at study enrollment. Extreme body mass index (BMI) before pregnancy (i.e., <14 or >40 kg/m^2^), implausible energy intake (lower or upper 2.5%), or no data on exposure, outcome, or other critical variables were criteria for exclusion. A total of 84,811 women were eligible for this analysis (Figure 1).

At the time of study enrollment, each woman was asked “how fast is your eating speed” with no specific guidance. Candidate responses were “very slow”, “relatively slow”, “medium”, “relatively fast”, or “very fast”. A validation study regarding eating speed in this population has not been performed, but there was evidence that self-reported eating speed showed good agreement with that reported by a friend [20]. The first two categories (“very slow” and “relatively slow”) were grouped as “slow” because of a low number of women in the “very slow” category (2.3%). Dietary assessment was performed using a semi-quantitative food frequency questionnaire that was used and validated in another cohort study [21]. Nutrient intakes were calculated based on the Japan Standard Tables of Food Composition (5th Revised Edition).

The first incidence of GDM was the outcome of interest. GDM was diagnosed when two or more values during the 75 g oral glucose tolerance test were greater than the cutoff levels: fasting plasma glucose ≥ 5.5 mmol/L (100 mg/dL), 1 h value ≥ 10.0 mmol/L (180 mg/dL), and 2 h value ≥ 8.3 mmol/L (150 mg/dL) [22]. GDM cases were identified using medical record transcripts, which were completed after delivery by physicians, research coordinators, nurses, or midwives.

Age-adjusted means and proportions of the pregnant women’s characteristics were calculated based on eating speed. Women who reported either very slow or relatively slow eating speed were treated as the reference group, and logistic regression was used to obtain the odds ratio (OR) and 95% confidence interval (CI) of GDM for other groups by comparing with the reference group. All ORs were age-adjusted in a basic model (model 1). We also adjusted for smoking status (never, past, or current smoker), drinking status (never, past, or current drinker), education level (middle school, high school, junior or specialized training college, or university or higher), occupation (15 categories), household income (nine categories), history of depression (yes or no), history of polycystic ovarian syndrome (yes or no), history of having macrosomia babies (yes or no), marital status (married, divorced, widowed, or other), parity (0, 1, 2, or ≥3), gestational weight gain (quintile), and physical activity (quintile) in model 2. In model 3, we further adjusted for dietary factors including white rice, seafood, meat, egg, coffee, green tea, milk, chocolate, soy isoflavones, dietary fiber, dietary magnesium, and dietary fat, and total energy intake (all quintiles). Pre-pregnancy BMI was adjusted in an additional model. To examine whether being overweight (pre-pregnancy BMI ≥25 kg/m^2^) could mediate the association between eating speed and GDM, and we also conducted a mediation analysis by treating being overweight as a potential mediator. This analysis was performed to compute the proportion of excess risk of GDM that could be attributed to the mediator, i.e., overweight. The mediation analysis was adjusted for the same covariates in model 3. Additionally, we performed sensitivity analyses to test the robustness of the results by restricting the analysis in women who reported the same eating speed in a second survey during mid-late pregnancy (gestational age: quartile 1 = 24 weeks, median = 27 weeks, quartile 3 = 29 weeks). All analyses were carried out using SAS 9.4 (SAS Institute Inc., Cary, NC, USA). All *P*-values were two-sided, with *p* < 0.05 considered statistically significant.

The JECS protocol was reviewed and approved by the Ministry of the Environment’s Institutional Review Board on Epidemiological Studies and by the Ethics Committees of all participating institutions (No.100406001). Written informed consent was obtained from all participants.

## 3. Results

We documented 1902 GDM cases from singleton pregnant women during follow-up, with the majority diagnosed during mid and late pregnancy. Table 1 shows the baseline characteristics of women by self-reported eating speed. Overall, the proportions of women who reported slow, medium, relatively fast, and very fast eating speed were 17.8%, 41.1%, 35.5%, and 5.7%, respectively. Women reporting very fast eating speed, compared with those reporting slow eating speed, were older and more likely to have a higher BMI, greater gestational weight gain, and a higher level of physical activity, but were less likely to be a housewife, nulliparous, or have never smoked. Regarding dietary factors, very fast eaters appeared to have higher intakes of total energy, white rice, meat, coffee, green tea, dietary fat, magnesium, and isoflavones, but lower intake of milk.

Table 2 shows the ORs (95% CIs) of GDM by self-reported eating speed. Compared with those for women reporting slow eating speed, the age-adjusted ORs (95% CI) of GDM for women who reported medium, relatively fast, and very fast eating speed were 1.03 (0.90, 1.18), 1.07 (0.94, 1.23), and 1.28 (1.05, 1.58), respectively. Adjustment for demographic factors, lifestyle-related factors, and other risk factors (model 2) and further adjustment for dietary factors (model 3) yielded similar results. When further analyses were performed after the inclusion of pre-pregnancy BMI, the association was attenuated and became no longer significant. In the mediation analysis, we examined whether being overweight could be a mediator for the association between eating speed and GDM. After controlling for the same covariates in model 3, being overweight accounted for 64% of the excess risk of GDM associated with eating speed (*p* < 0.001), indicating that increased body fat may have largely mediated the association observed. 

Table 3 shows the ORs (95% CIs) of GDM by self-reported eating speed among 64,183 women who reported the same eating speed during early and late pregnancy. Overall, the association appeared to be somewhat stronger in this sensitivity analysis. After adjustment for all covariates including pre-pregnancy BMI, a very fast eating speed was independently associated with GDM (OR = 1.32; 95% CI: 1.03, 1.70).

## 4. Discussion

To our knowledge, this large prospective cohort study is the first study to investigate eating speed in relation to GDM risk. Women who reported very fast eating speed at recruitment (median: 12 weeks of gestation), compared with those reporting slow eating speed, had an increased incidence of GDM, which was independent of demographic, lifestyle-related, and dietary factors. The association was attenuated and no longer significant after further adjustment for pre-pregnancy BMI. The mediation analysis showed that being overweight accounted for 64% of the excess risk of GDM associated with eating speed.

Interestingly, in our sensitivity analysis, restricting for 64,183 women (75.7%) who reported the same eating speed during early and mid-late pregnancy, the association remained significant for women reporting very fast eating speed. One explanation for this result may be that changes in eating speed during pregnancy may have biased the association in the main analysis toward the null.

The central nervous system regulates appetite [23], and eating induces production of satiety hormones including cholecystokinin, peptide YY, and glucagon-like peptide-1, which are involved in appetite regulation [24,25]. Fast eating speed has been found to be associated with a lower level of satiety hormones [26], which may result in a delayed feeling of fullness and, thus, excess energy intake. In fact, in the present study eating speed was positively associated with energy intake (Table 1). A meta-analysis of 22 randomized controlled trials examining the effect of eating speed on energy intake also provided evidence that faster eating speed was associated with higher energy intake than slower eating speed [2].

Excess energy intake over the long term could lead to weight gain, and thereby to being overweight and obesity. A meta-analysis of cross-sectional studies showed a significant difference in BMI between individuals who ate quickly and those who ate slowly (mean difference: 1.78 kg/m^2^, 95% CI:1.53, 2.04) [1]. The same meta-analysis also found fast easting speed had a stronger association with higher prevalence of obesity (pooled OR 2.15, 95% CI: 1.84, 2.51) than slow eating speed. Prospective cohort studies, in which a temporal relationship can be established, also showed fast eating speed may be a risk factor for weight gain and obesity. For example, a cohort study of 1314 university students found, after 3 years of follow-up, that those who ate quickly had an increased risk of obesity compared with those who ate slowly [27].

The mechanisms underlying the association between eating speed and GDM are unclear. As mentioned above, fast eating speed was associated with excess energy intake and higher risk of obesity, leading to development of GDM. This was supported by our mediation analysis, showing that pre-pregnancy BMI was a potential mediator in the association between eating speed and GDM. Evidence also showed eating quickly was associated with insulin resistance: in a cross-sectional study of 2704 men and 761 women, eating speed was positively associated with insulin resistance, which was independent of age, energy intake, and lifestyle factors [28]. However, the association persisted only in men when BMI was further adjusted.

We are aware of one prospective cohort study examining the effect of eating speed on the incidence of type 2 diabetes. In that study of 2050 men, after 7 years of follow-up, fast eating speed was associated with an increased risk of type 2 diabetes (relative risk = 1.97 [95% CI: 1.10–3.55]) [6]. However, that association disappeared after further adjustment for baseline BMI, which was in line with our findings in the main analysis.

The main strengths of the present study include a large sample size and a prospective design. Limitations, however, should also be mentioned. First, measurement of eating speed was based on responses to a self-reported questionnaire but not based on objective measurement, such as the size of the meal and time spent on the meal. We could not rule out the risk of measurement errors, which could bias the association toward the null. Of note, a previous study in a Japanese population showed good agreement between self-reported and friend-reported eating speed [20]. Second, although we carefully controlled for known risk factors and potential confounding factors, it was uncertain to what extent residual confounding due to unmeasured factors may have influenced our results. For example, data on family history of diabetes were absent, though this factor was unlikely to be associated with the exposure (eating speed). Third, GDM incidence was relatively lower in this population compared with that in other populations. We have reported possible explanations elsewhere [29]. Briefly, it may be explained by the lower pre-pregnancy BMI in this population, by different diagnosis criteria used, and by the exclusion of about 800 women with a history or current diagnosis of GDM at the time of study enrollment. Fourth, dietary habits including eating speed may change in pregnant women. In our sensitivity analysis, restricting for women who reported the same eating speed during early and mid-late pregnancy, the association remained significant, indicating changes in eating speed during pregnancy may have biased the association in the main analysis toward the null.

## 5. Conclusions

In conclusion, the present large prospective cohort study suggested that very fast eating speed was associated with increased incidence of GDM, which may be largely mediated by increased body fat. Further studies among other populations are warranted.

## Figures and Tables

**Table 1:** Characteristics of 84,811 pregnant women according to self-reported eating speed.

	Slow	Medium	Relatively Fast	Very Fast	*p*
No. of participants	15,061	34,857	30,080	4813	
Age, years	29.8	30.7	31.0	31.5	<0.001
BMI before pregnancy, kg/m^2^	20.6	21.0	21.5	21.8	<0.001
Gestational weight gain, kg	10.1	10.3	10.3	10.5	0.03
University or higher education, %	23.2	20.8	22.4	22.4	<0.001
Family income < 2 million/y, %	5.3	5.1	4.6	5.2	<0.001
Housewife, %	28.2	29.0	26.3	23.0	<0.001
Married, %	94.6	95.3	95.5	94.9	<0.001
Nulliparous, %	48.3	39.2	40.6	42.4	<0.001
History of macrosomia baby, %	0.3	0.5	0.5	0.6	0.006
Never smoker, %	61.1	59.7	56.9	52.4	<0.001
Never drinker, %	54.4	54.5	56.3	56.0	<0.001
Depression, %	3.7	2.7	2.8	3.9	<0.001
Polycystic ovarian syndrome, %	2.4	2.0	2.4	2.2	0.005
Physical activity, Met∙h/day	3.6	3.9	3.9	4.6	<0.001
Total energy, kcal/day	1752	1750	1798	1880	<0.001
White rice, g/day	272.9	281.3	292.5	305.2	<0.001
Seafood, g/day	37.5	37.5	38.2	39.3	<0.001
Meat, g/day	68.9	69.8	74.2	81.0	<0.001
Egg, g/day	29.6	30.4	31.9	34.5	<0.001
Coffee, g/day	101	104	111	118	<0.001
Green tea, g/day	165	161	165	182	<0.001
Milk, g/day	135	127	123	122	0.003
Total dietary fat, g/day	58.6	58.2	60.1	63.6	<0.001
Magnesium, mg/day	233	233	238	246	<0.001
Total dietary fiber, g/day	11.0	11.0	11.2	11.5	<0.001
Chocolate, g/day	6.2	5.6	5.8	6.4	<0.001
Isoflavones, mg/day	30.6	31.2	31.6	32.8	<0.001

Values are means unless otherwise specified. *P* values were calculated using ANOVA or the chi-square test for continuous or categorical variables, respectively. BMI: body mass index.

**Table 2:** Self-reported eating speed and risk of gestational diabetes mellitus among 84,811 women.

	Slow	Medium	Relatively Fast	Very Fast
No of participants	15,061	34,857	30,080	4813
No of cases	298	766	699	139
Model 1	1.00	1.03 (0.90, 1.18)	1.07 (0.94, 1.23)	1.28 (1.05, 1.58)
Model 2	1.00	1.08 (0.94, 1.24)	1.13 (0.99, 1.30)	1.35 (1.10, 1.66)
Model 3	1.00	1.08 (0.94, 1.24)	1.11 (0.97, 1.28)	1.29 (1.05, 1.59)
Model 3 + pre-pregnancy BMI	1.00	1.04 (0.90, 1.19)	1.01 (0.88, 1.16)	1.14 (0.93, 1.41)

Model 1: adjusted for age; Model 2: Model 1 and further adjusted for education, occupation, household income, smoking, drinking, history of depression, history of polycystic ovarian syndrome, history of macrosomia babies, parity, gestational weight gain, physical activity; Model 3: Model 2 and further adjusted for intakes of white rice, seafood, meat, egg, coffee, chocolate, green tea, milk, soy isoflavone, magnesium, total dietary fat, total dietary fiber, and total energy. BMI: body mass index.

**Table 3:** Self-reported eating speed and risk of gestational diabetes mellitus among 64,183 women who reported the same eating speed during early and mid-late pregnancy.

	Slow	Medium	Relatively Fast	Very Fast
No. of participants	10,783	26,520	23,777	3103
No. of cases	206	578	541	97
Model 1	1.00	1.07 (0.91, 1.25)	1.09 (0.93, 1.29)	1.45 (1.13, 1.85)
Model 2	1.00	1.11 (0.95, 1.31)	1.16 (0.98, 1.37)	1.55 (1.21, 1.99)
Model 3	1.00	1.17 (0.95, 1.31)	1.15 (0.97, 1.35)	1.50 (1.16, 1.92)
Model 3 + pre-pregnancy BMI	1.00	1.07 (0.91, 1.26)	1.04 (0.88, 1.23)	1.32 (1.03, 1.70)

Model 1: adjusted for age; Model 2: Model 1 and further adjusted for education, occupation, household income, smoking, drinking, history of depression, history of polycystic ovarian syndrome, history of macrosomia babies, parity, gestational weight gain, physical activity; Model 3: Model 2 and further adjusted for intakes of white rice, seafood, meat, egg, coffee, chocolate, green tea, milk, soy isoflavone, magnesium, total dietary fat, total dietary fiber, and total energy.
